# miRTarVis: an interactive visual analysis tool for microRNA-mRNA expression profile data

**DOI:** 10.1186/1753-6561-9-S6-S2

**Published:** 2015-08-13

**Authors:** Daekyoung Jung, Bohyoung Kim, Robert J Freishtat, Mamta Giri, Eric Hoffman, Jinwook Seo

**Affiliations:** 1 Department of Computer Science and Engineering, Seoul National University, 1, Gwanak-ro, Gwanak-gu, Seoul, South Korea; 2 Department of Radiology, Seoul National University Bundang Hospital, 300 Gumi-dong, Bundang-gu, Seongnam-si, Gyeonggi-do, South Korea; 3 Division of Emergency Medicine, Children's National Medical Center, Washington, D.C., USA; 4 Center for Genetic Medicine Research, Children's National Medical Center, Washington, D.C., USA; 5 Department of Integrative Systems Biology, George Washington University, 111 Michigan Avenue, NW, Washington, D.C., 20010-2970, USA

**Keywords:** MicroRNA, mRNA, Visualization, Expression profile, Target prediction

## Abstract

**Background:**

MicroRNAs (miRNA) are short nucleotides that down-regulate its target genes. Various miRNA target prediction algorithms have used sequence complementarity between miRNA and its targets. Recently, other algorithms tried to improve sequence-based miRNA target prediction by exploiting miRNA-mRNA expression profile data. Some web-based tools are also introduced to help researchers predict targets of miRNAs from miRNA-mRNA expression profile data. A demand for a miRNA-mRNA visual analysis tool that features novel miRNA prediction algorithms and more interactive visualization techniques exists.

**Results:**

We designed and implemented *miRTarVis*, which is an interactive visual analysis tool that predicts targets of miRNAs from miRNA-mRNA expression profile data and visualizes the resulting miRNA-target interaction network. *miRTarVis *has intuitive interface design in accordance with the analysis procedure of load, filter, predict, and visualize. It predicts targets of miRNA by adopting Bayesian inference and MINE analyses, as well as conventional correlation and mutual information analyses. It visualizes a resulting miRNA-mRNA network in an interactive Treemap, as well as a conventional node-link diagram. miRTarVis is available at http://hcil.snu.ac.kr/~rati/miRTarVis/index.html.

**Conclusions:**

We reported findings from miRNA-mRNA expression profile data of asthma patients using miRTarVis in a case study. miRTarVis helps to predict and understand targets of miRNA from miRNA-mRNA expression profile data.

## Background

All organisms use selective gene transcription of mRNAs to carry out biological functions. Increasingly, it is recognized that regulatory RNAs (microRNA (miRNA)), long non-coding RNA (lnRNA) play key roles in regulating the stability and translation of existing pools of mRNAs in any cell. Thus, understanding the regulation of the genome requires the integration of mRNA, as well as the regulatory RNAs targeting and regulating those mRNAs. Of the regulatory RNAs, miRNAs are the best characterized and studied. MiRNAs are short highly processed oligonucleotides (approx. 22 nt) that carry out post-transcriptional regulation of target mRNAs through either degradation of the target mRNA or inhibition of protein translation [1].

The specific mRNA targets for any specific miRNA can be derived bioinformatically through miRNA-mRNA sequence alignment and evolutionary conservation of the target mRNA sequence. For example, miRNA target prediction algorithms such as TargetScan [2] or miRanda [3] predict targets of miRNAs. The potential interactions between any miRNA-mRNA pair require experimental validation, typically through reporter constructs.

Recent prediction algorithms used miRNA-mRNA expression profile data. Microarray method has been prevalent before deep sequencing method becomes popular recently for miRNA-mRNA expression profiling. As deep sequencing methods become widespread, whole genome miRNA-mRNA expression profile data become widely available. Accordingly, some algorithms exploited miRNA-mRNA expression profile data to search for targets of miRNAs. Bioinformaticians also introduced web-based tools [4-6] to integrate miRNA-mRNA expression profile data with sequence-based miRNA target prediction algorithms.

However, those tools are limited in supporting rich exploratory analysis of miRNA-mRNA expression profile data. For example, enabling dynamic queries and providing relevant biological information on demand in the visualizations are among the much-expected features. We believe that more work is required in visualization and interaction design of visual analysis tools for miRNA-mRNA expression profile data. Given that both miRNA and mRNA expression datasets are multidimensional, searching for mRNA targets requires integrative analysis of the two heterogeneous multidimensional datasets. To obtain more improved accuracy of such integrative multidimensional data analysis, interactive visual analysis tools for miRNA-mRNA expression profile data should help researchers:

• predict miRNA-target interactions by integrating miRNA-mRNA expression profile datasets and

• understand the structure of miRNA-mRNA interaction network.

We developed *miRTarVis*, an interactive visual analysis tool that predicts targets of miRNAs from miRNA-mRNA expression profile data and visualizes miRNA-target interaction network that is derived from the prediction. Based on observations and interviews, we first defined a common analysis pipeline for miRNA-mRNA expression profile data, and then we designed the interface of *miRTarVis *based on the analysis pipeline. *miRTarVis *provides prediction algorithms that are based on both sequence and expression profile data. *miRTarVis *is the first visual analysis tool that applies GenMiR++ [7], a Bayesian inference model, and MINE (Maximal Information-based Nonparametric Exploration) analysis [8], a new technique that finds highly associated pairs from multidimensional data, to predict targets of miRNAs from miRNA-mRNA expression profile data. *miRTarVis *can visualize a resulting bipartite miRNA-target regulatory network in interactive node-link diagram and Treemaps. The Treemap is a unique feature of *miRTarVis*, and it is expected to outperform a node-link diagram visualization when miRNA-target interactions are overcrowded. We proved the efficacy of *miRTarVis *in a case study by applying it to human miRNA-mRNA expression profile data.

## Related works

### MiRNA target prediction algorithms

It is known that miRNAs and their target mRNAs usually have complementary sequences in the seed region of the miRNA and 3' UTR of the mRNA. Some target prediction algorithms predicted targets of miRNAs using sequence-based rules.

**Figure 1:**
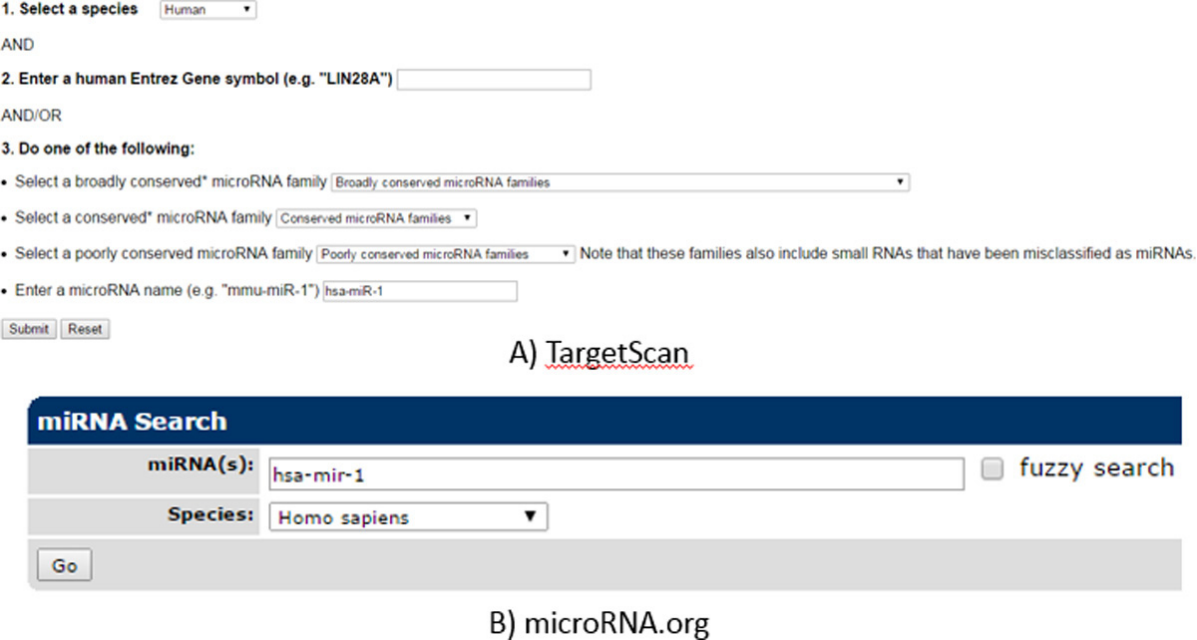
**Interfaces of TargetScan and microRNA.org**.

TargetScan [2] predicts targets of miRNAs by searching conserved 8mer or 7mer sites that match the seed region of each miRNA. It provides a web service that gives a list of targets for a given miRNA (http://www.targetscan.org). The website has simple forms through which users can submit queries (Figure 1). For a given miRNA, the website returns a list of predicted target genes of the miRNA in a text table. It also shows how a miRNA and its target mRNA are aligned to each other.

microRNA.org [9] searches for targets of a miRNA by using the miRanda algorithm [3]. It uses a weighted complementary sequence score between a miRNA and a mRNA, giving a higher score for complementarity in a miRNA seed region and a lower score for complementarity in other regions. As a result, it predicts those mRNAs that match not only in the 5' end seed region of a specific miRNA but also in the 3' end of a miRNA as a possible target of the miRNA. The microRNA.org web service provides a list of predicted targets sorted by the mirSVR score [10] for a given miRNA and shows alignment between a miRNA and its targets.

Both TargetScan and microRNA.org provide web services that give back a list of predicted targets for a given miRNA. However, their interfaces take only one miRNA at a time, and thus, it is difficult to query multiple miRNAs at once, which in turn makes it difficult to study interactions among many miRNA-mRNA pairs. It is also not straightforward to integrate the prediction result of TargetScan or microRNA.org with miRNA-mRNA expression profile data. A general analysis procedure for miRNA-mRNA expression profile data is to find some significant (e.g., differentially expressed) miRNAs and mRNAs from miRNA-mRNA expression profile data and search for miRNA-target interactions among them based on a prediction algorithm. However, the web interfaces of sequence-based prediction algorithms are not suitable for integrating miRNA-mRNA expression profile data with the prediction algorithms. Other miRNA prediction web services [11,12] have the same problems.

There were other types of algorithms that searched for miRNA targets from miRNA-mRNA expression profile data. GenMiR++ [7] used a Bayesian inference model to search for targets of miRNAs from miRNA-mRNA expression profile data. Hoctar [13] searched for anti-correlated miRNA-mRNA pairs to predict targets of a miRNA. Zhang et al. [14] suggested a mathematical framework that searches for miRNA-gene regulatory modules by integrating prior information of miRNA target prediction with miRNA-gene expression profile. It is reported that these algorithms increased the specificity of sequence-based target prediction algorithms. miRTarVis adopted the GenMiR++ algorithm among these algorithms because the Bayesian inference model can reveal causal relationships between miRNAs and their targets.

### Analysis tools for miRNA-mRNA expression profile data

Some tools helped miRNA researchers predict targets of miRNAs and visualize a resulting miRNA-target interaction network by integrating miRNA-mRNA expression profile data with prediction algorithms. MicroRNA and mRNA integrated analysis (MMIA) [4] is a web-based tool that integrates miRNA-mRNA expression profile data with three different sequence-based prediction algorithms. It first searches for differentially up-regulated miRNAs. Next, it searches for their target mRNAs among differentially down-regulated mRNAs by sequence-based prediction algorithms. MMIA also performs gene set analysis and other optional analyses. MMIA shows the analysis results in a simple text table without any visualizations.

miRConnX [5] is a web-based tool that creates a regulatory network from miRNA-mRNA expression profile data and visualize the network. It creates two types of networks: static and dynamic. Static network is created from prior knowledge (i.e., sequence-based miRNA prediction algorithms) regardless of the input data. It consists of transcription factors (TFs)-gene and miRNA-gene predicted or validated associations derived from previous studies. miRConnX creates a dynamic network from input miRNA-mRNA expression profile data. A user can choose an association measure among Pearson, Spearman, or Kendall correlation coefficients. miRConnX combines the static and dynamic networks into an integrated network. It shows the resulting network in a node-link diagram.

Magia [6] predicts targets of miRNAs from miRNA-mRNA expression profile data in a manner similar to miRConnX, but it adopts mutual information, as well as Spearman and Pearson correlation coefficients. Magia also visualizes miRNA-mRNA-TF regulatory networks in a node-link diagram. The number of links in the node-link diagram is limited up to 200.

MMIA, miRConnX, and Magia integrated miRNA-mRNA expression profile data with sequence-based prediction algorithms. MMIA used two-sample expression profile data, whereas miRConnX and Magia used multisample expression profile data. MMIA could search for differentially expressed miRNAs and mRNAs before applying prediction algorithms. miRConnX and Magia do not search for differentially expressed miRNAs and mRNAs using multisample expression profile. Instead, they predict targets of miRNAs by searching for highly associated miRNA-mRNA pairs from predicted miRNA-mRNA pairs. MMIA presented a resulting miRNA-target interaction network in a text table, and miRConnX and Magia presented it in a simple node-link diagram.

## Methods

### Design goals and rationales

At the first stage of our iterative design process, we tried to define a common analysis pipeline for miRNA-mRNA expression profile data. We observed researchers analyze miRNA data and conducted informal interviews with them. We identified four major analysis steps in their analysis process for miRNA-mRNA expression data, which constitute the analysis pipeline. Our analysis pipeline consists of the following:

*1. load*: Load miRNA-mRNA expression profile data.

*2. filter*: Gilter miRNA-mRNA expression profile data to leave only significant miRNAs and mRNAs for further analysis.

*3. predict*: Predict miRNA-target interactions by sequence-based prediction algorithms and search for highly associated miRNA-mRNA pairs in expression profile data by data mining or machine learning techniques.

*4. visualize*: Visualize the resulting miRNA-target network to help researchers understand the network structure and biological implication of the network.

After deriving the analysis pipeline, our long-term design collaborative design process with biomedical researchers led us to the following design goals of our visual analysis tool for miRNA-mRNA expression profile data. It should help users:

1. Aanalyze miRNA-mRNA expression profile data of various types based on the analysis pipeline

2. Improve miRNA target prediction accuracy by integrating multiple target prediction algorithms

3. Csomprehend the resulting miRNA-mRNA interaction network through interactive visualizations

To achieve the design goals, we designed and implemented our visualization tool based on the following design rationales:

1. Provide a user interface based on the analysis pipeline.

2. Support various types of miRNA-mRNA expression profile data.

3. Support interactive filtering of less significant or erroneous miRNAs and mRNAs for better prediction accuracy.

4. Integrate diverse prediction algorithms, including novel prediction algorithms, for more accurate prediction results.

5. Present analysis results in intuitive visualizations.

6. Support dynamic queries through intuitive user interactions to help users search biological findings.

### Overview of unique features of *miRTarVis*

The user interfaces of *miRTarVis *are designed based on the analysis pipeline for miRNA-mRNA expression profile data. The design provides good affordance that enables users to naturally follow the analysis pipeline step by step. We adopted an accordion metaphor to organize the four steps of the analysis pipeline with the load step at the top followed by filter, predict, and visualize steps.

*miRTarVis *provides users more flexibility in preparing input miRNA-mRNA expression profile data. *miRTarVis *can accept both two-sample and multisample miRNA-mRNA expression profile data. It also accepts data that only consist of fold change and p-value without underlying expression data. It also directly accepts TCGA (The Cancer Genome Atlas) miRNA-mRNA expression profile data.

*miRTarVis *supports filtering functions most appropriate for each input data type. For two-sample expression profile data, p-value and fold change of each mRNA and miRNA are calculated automatically on loading, and users can filter data by p-value and fold change. For multisample expression profile data, users can filter out poorly expressed (e.g., most expression levels are zero) miRNAs and mRNAs.

*miRTarVis *is the first tool that applies the MINE analysis [8] to search for targets of miRNAs from miRNA-mRNA expression profile data. The MINE analysis is adopted to support finding more general relationships because it can search for not only conventional linear relationships but also nonlinear and nonfunctional relationships. *miRTarVis *also supports finding causal relationships between miRNAs and their targets from miRNA-mRNA expression profile data by adopting a Bayesian inference modeling analysis (GenMiR++ [7]). miRTarVis provides an interactive web interface for GenMiR++ to improve its usability from the original command-line interface available in Matlab. *miRTarVis *also supports correlation analysis and mutual information analysis.

*miRTarVis *is the first tool that adopts a Treemap to show a resulting miRNA-target regulatory network in such a way that a miRNA node encloses its target mRNAs in a Treemap layout. In this way, the Treemap is more effective than the traditional node-link diagram when the network is overcrowded by a large number of miRNA-target interactions. *miRTarVis *also visualizes a miRNA-target network in an improved node-link diagram where multiple miRNA-target nodes are connected to a miRNA node in a radial layout. Users can navigate the diagram interactively for closer inspection of an interesting miRNA or mRNA. Users can also move miRNA or mRNA nodes to a better position. This enhanced interactivity can help users understand the structure of a miRNA-target network and can make a node-link diagram more suitable for publication. *miRTarVis *also helps researchers access relevant detailed information for miRNAs and mRNAs by linking a corresponding website that contains the information upon a mouse click. We will discuss this in detail later when we explain the visualization of miRNA-target network.

### Prediction of miRNA targets

In the *predict *step, *miRTarVis *searches for miRNA-mRNA target interactions among all remaining miRNA-mRNA pairs from the previous step. *miRTarVis *uses prediction algorithms that are based on both sequence and expression profile data. *miRTarVis *supports two sequence-based prediction algorithms, TargetScan and microRNA.org, which are two of the most cited miRNA target prediction algorithms. *miRTarVis *supports four techniques for prediction algorithms based on expression profile data, and these techniques are correlation analysis (a user can choose to use Pearson or Spearman coefficient), mutual information analysis, Bayesian inference model analysis (GenMiR++), and MINE analysis. Given that prediction algorithms based on expression profile data require expression level at each sample, *miRTarVis *does not support this type of prediction for the third data type of *p-value and fold change*. As discussed earlier, *miRTarVis *is the first tool that can apply Bayesian inference (GenMiR++) and MINE analyses.

Prediction algorithms based on expression profile data calculate scores that represent the intensity of association for each miRNA-target pair. *miRTarVis *allows users to set a threshold value to filter miRNA-mRNA interactions by their score when conducting the predictions. *miRTarVis *can also set the number of resulting miRNA-target interactions for prediction. In this case, *miRTarVis *searches for the top high-scored miRNA-target interactions.

miRTarVis can filter miRNA-mRNA interactions by their fold change direction as well. For example, predicted miRNA-mRNA interactions where miRNA is up-regulated and mRNA is down-regulated are biologically more significant because miRNAs down-regulate their target mRNAs. To enable diverse filtering by fold change direction, *miRTarVis *provides four search options for fold change direction of miRNA-mRNA interactions in prediction: up-regulated miRNA and down-regulated mRNA, down-regulated miRNA and up-regulated mRNA, oppositely regulated (union of the first and the second options), and all pairs. If prediction is confined to miRNA-mRNA pairs that consist of up-regulated miRNA and down-regulated mRNAs or pairs that consist of down-regulated miRNA and up-regulated mRNAs, the accuracy of prediction could be improved. Therefore, miRTarVis enables users to use these options to improve the accuracy of their miRNA target prediction.

### Visualization of miRNA-target network

In the *visualize *step, *miRTarVis *generates a regulatory network between miRNAs and their targets from the target prediction result in the previous *predict *step and visualizes the resulting bipartite graph both in a node-link diagram and a Treemap.

To achieve the aforementioned design goals of *miRTarVis*, visualizations and interactions in *miRTarVis *should be designed to help users to understand the predicted miRNA-target network effectively. More specifically, *miRTarVis *should provide the following functionalities:

• Reveal topological structures in the network

• Highlight mRNAs that are regulated commonly by multiple miRNAs in the network

• Provide information of particular miRNAs and mRNAs on demand

• enable users to go back to the previous step and adjust parameters to update the miRNA-target interaction network

We adopt a node-link diagram and a Treemap visualization to achieve the design goals. The node-link diagram equipped with improved layout algorithms can help reveal the overall topological structures of a miRNA-target interaction network. The Treemap visualization can better reveal mRNA targets for each miRNA in a more space-efficient manner without occlusion.

**Figure 2:**
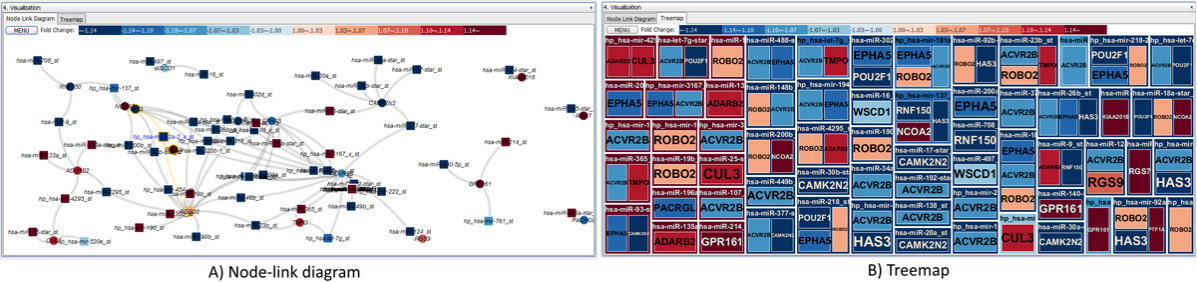
**Example of node-link diagram and Treemaps for a miRNA-mRNA regulatory network**. Red and blue represent up- and down-regulated fold changes, respectively. Color saturation represents the intensity of a fold change.

**Figure 3:**
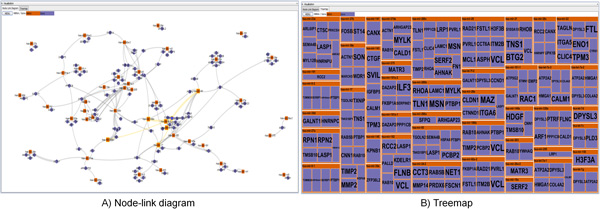
***miRTarVis *visualizing a miRNA-target interaction network from TCGA breast cancer data in node-link diagram and Treemaps**.

In a node-link diagram (Figures 2A and 3A), a layout algorithm determines how it presents the shape of the network. One of the most popular layout algorithms is force-directed layout algorithm. It determines the positions of network nodes by calculating the repulsive and attractive forces between nodes. However, it does not consider the topological structure of the graph, which results in much occlusion between links when the network consists of a large number of nodes. The miRNA-target prediction network is a bipartite graph where there are more mRNA (target) nodes than miRNA nodes, because one miRNA regulates multiple mRNAs simultaneously. Most target mRNAs in the network are also predicted to be regulated by only one miRNA. Based on these observed characteristics of miRNA-target networks, we decided to employ layout algorithms that consider the topological structure of the miRNA-target network. The ISOM [15] and KK layouts [16] are thus introduced in *miRTarVis *in addition to the popular force-directed layout and circular layout algorithms. However, the ISOM layout that considers only topological relationships places the mRNA nodes connected to a miRNA node in the same position. To address this problem, we improved the algorithm by scattering such mRNA nodes around the miRNA node in a circular manner.

*miRTarVis *uses color as a unique visual cue to help users easily recognize whether a predicted miRNA-mRNA interaction is supported by input experimental miRNA-mRNA expression profile data. For two-sample data, the fold change value is color coded: up-regulated miRNAs and mRNAs in red and down-regulated miRNAs and mRNAs in blue. The intensity of fold change is represented by color saturation, and darker color means higher degree of fold change (Figure 2). A miRNA down-regulates the expression levels of its target mRNAs. Therefore, a user can easily grasp whether the prediction is supported by the input data or not by comparing the colors of the ends of a link. For multisample miRNA-mRNA expression profile data, *miRTarVis *represents miRNAs in orange and mRNAs in dark blue (Figure 3). If a part of a network looks interesting, a user can navigate (zoom and pan) around a specific miRNA with simple mouse wheel interaction in the node-link diagram.

*miRTarVis *can also visualize a miRNA-target regulatory network using a Treemap (Figures 2B and 3B). One characteristic of miRNA-target interaction networks is that one miRNA node is connected to many target mRNA nodes. We could convert the network into a two-level hierarchy where a miRNA is a parent of target mRNAs by exploiting this characteristic, and a Treemap visualization can be used to represent the network. In the tree, leaf nodes represent mRNA nodes, and their parent nodes represent miRNA nodes that have link with the mRNAs. Therefore, if one mRNA node has links with multiple miRNA nodes, the mRNA node occurs multiple times in the Treemap visualization. In the original Treemap, the area encodes an attribute of the data. However, in our Treemap visualization, all mRNA nodes have the same area. Therefore, the size of a miRNA node reflects the number of its target mRNAs in the network.

Compared with a node-link diagram, a Treemap visualization can represent a complex regulatory network without occlusion among links and nodes, especially when there are too many links crossing each other in a node-link diagram. Given that a Treemap visualization is much more space efficient than a node-link diagram, more screen space can be devoted to showing gene symbol names without occlusion; these names serve as important information for biologists to understand the biological functions of mRNAs in a miRNA-target interaction network. For example, in Figure 3, a miRNA-target interaction network is visualized in both Treemap and node-link diagram. Identifying targets of a miRNA in the middle part of the node-link diagram is more difficult than in Treemap because there is not much room for showing gene symbols for target mRNAs without occlusion.

**Figure 4:**
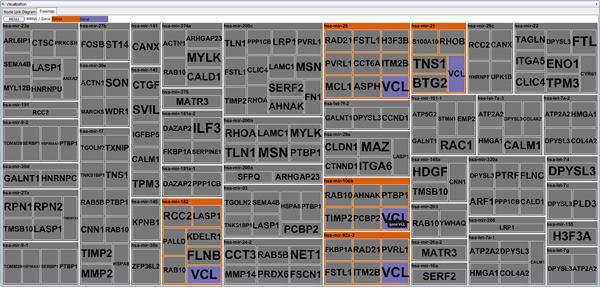
**Identical mRNAs highlighted in the Treemap of *miRTarVis *while fading out other miRNAs and mRNAs**.

However, the Treemap visualization has a disadvantage of not showing the overall structure of the network. *miRTarVis *represents all miRNAs as top-level nodes and all their targets as their child nodes. As a result, if multiple miRNAs have a common target mRNA, the mRNA node appears multiple times in the Treemap, and there is no affordance to imply whether an mRNA is connected to multiple miRNAs. We tried to resolve this problem by interactively highlighting all nodes representing a selected mRNA node in our Treemap visualization (Figure 4).

In the *visualize *step, users can efficiently explore the resulting miRNA-mRNA regulatory network by using the two visualizations. If the result is not satisfactory, *miRTarVis *enables users to go back to *filter *or *predict *steps to change the parameters of filtering or prediction for a better result. Through this iterative procedure, *miRTarVis *can help users efficiently narrow down to more important and interesting miRNA-target interactions.

### UI and functions of *miRTarVis*

**Figure 5:**
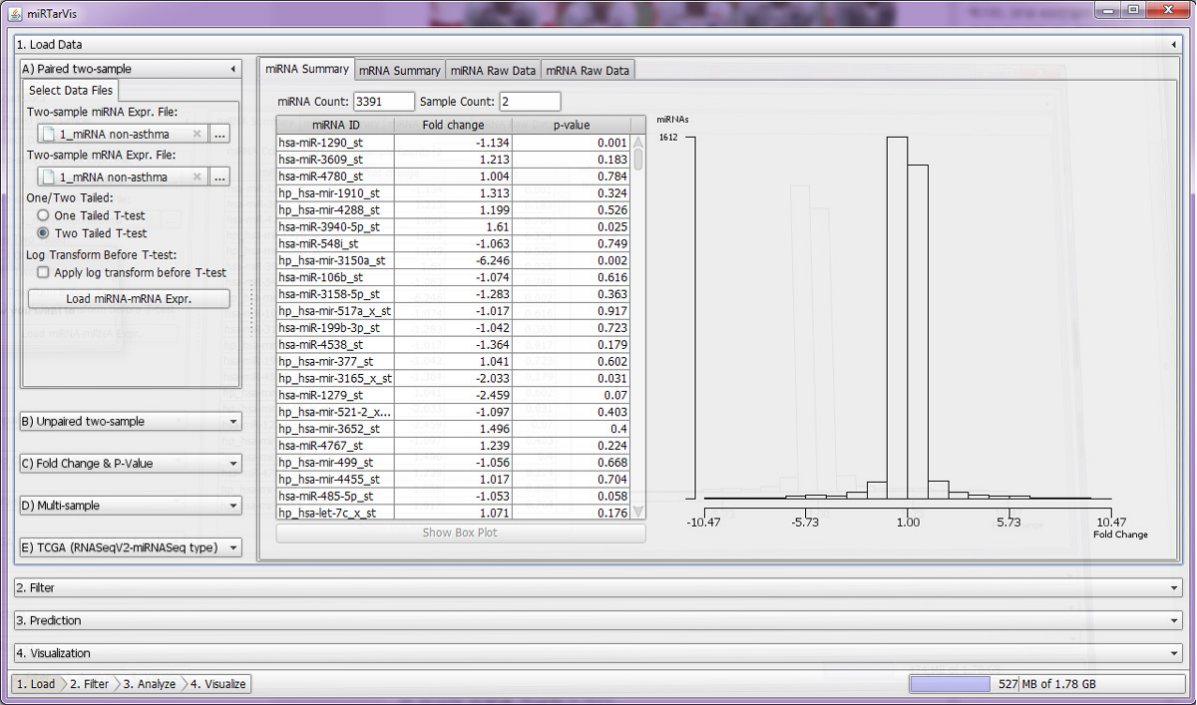
***miRTarVis *interface in a simple foldable accordion metaphor**. In this figure, the *predict *menu is selected, and other menus are folded.

We designed *miRTarVis *based on the *load-filter-predict-visualize *pipeline (Figure 5). We adopted a simple step-by-step foldable accordion metaphor, where only one selected menu item is open while all others are collapsed. This is more intuitive to users because the order of the menu items matches the order of the analysis procedure. Through this foldable accordion interface, users can focus on the current step of the procedure while using the screen space more effectively even in a small-size screen (down to a screen resolution of 1280 × 720).

**Figure 6:**
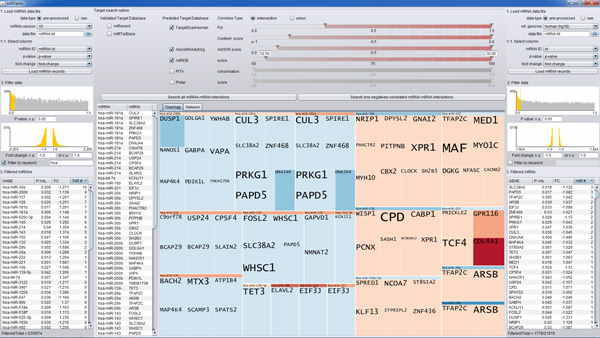
**First design of *miRTarVis***. Interfaces of load, filter, prediction, and visualization are all placed in one window.

At the early stage of our iterative design process, we designed miRTarVis to perform all procedures in one large screen [17] (Figure 6). In that design, all information appeared simultaneously: miRNA expression profile data on the left, mRNA expression profile data on the right, prediction algorithm on the top, and main visualization at the center. However, that design lacked guidance or affordance about how to perform the analysis procedure and required a large screen space. Therefore, we adopted a foldable accordion metaphor reflecting the analysis pipeline.

**Figure 7:**
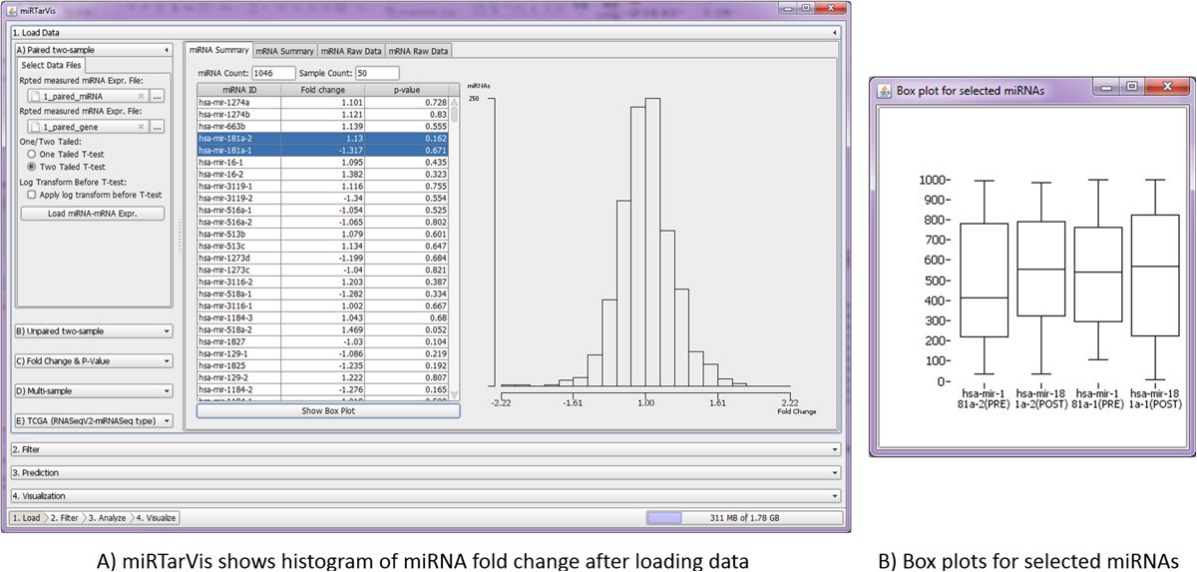
**Load menu**. *miRTarVis *shows loaded data in a table, histogram, and box plot.

There are four big menus: load data, filter, predict, and visualize. In the load data menu (Figure 7), miRTarVis can load one of five types (paired two-sample, unpaired two-sample, p-value and fold change, multisample, and TCGA) of miRNA-mRNA expression profile data.

In the *filter *menu, *miRTarVis *can select only significant miRNAs and mRNAs that will serve as a search space in the next *predict *step. *miRTarVis *provides different filtering options for two-sample and multisample type data: filtering by p-value or fold change for the former and filtering by average expression level for the latter. *miRTarVis *shows a histogram of fold change for two-sample type data and a histogram of average expression level for multisample type data.

**Figure 8:**
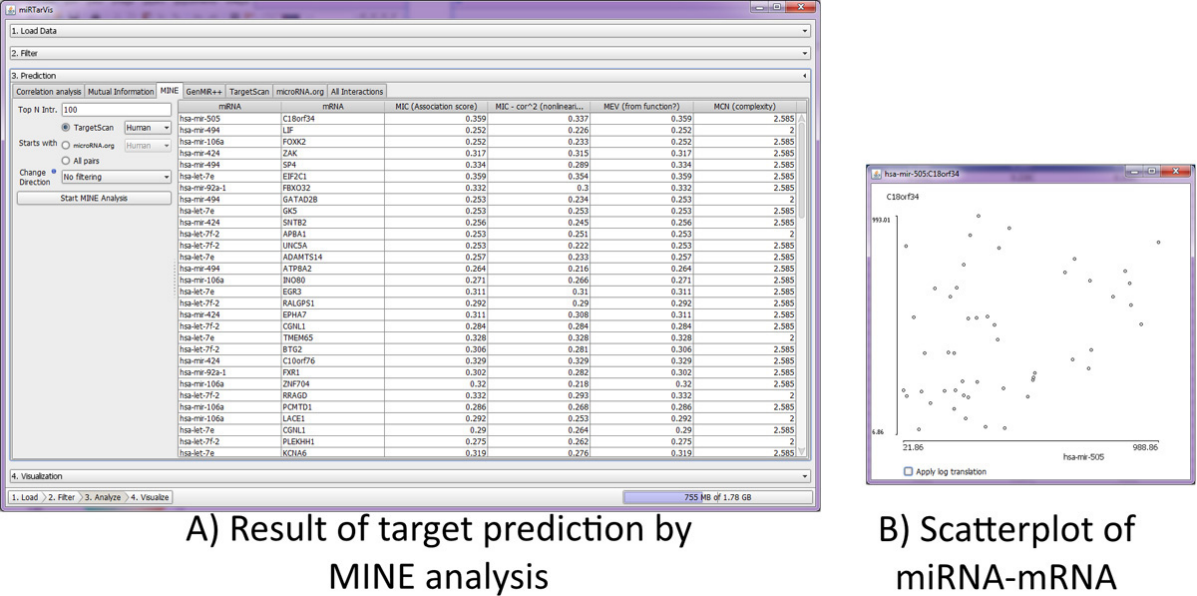
**Prediction menu**. *miRTarVis *shows target prediction results by MINE analysis in a table.

In the *predict *menu (Figure 8A), *miRTarVis *searches for targets of miRNAs by multiple algorithms. The *predict *menu uses a tab interface where each tab is dedicated to a prediction algorithm. When a user selects a certain prediction algorithm in a tab, the interface for choosing parameters for the selected algorithm appears on the left. *miRTarVis *searches for miRNA-target pairs among miRNAs and mRNAs that survived from the previous *filter *step. After finishing the prediction process, *miRTarVis *shows the predicted miRNAs and their targets in a table on the right. *miRTarVis *can show a scatterplot of the expression levels of the predicted miRNA-target interaction (Figure 8B). This scatterplot shows the relationship between miRNA and mRNA directly. In the last tab (named "All interactions"), *miRTarVis *summarizes all the miRNA-target interactions found so far in a table. In the *All interactions *tab, miRTarVis can intersect prediction results of different user-selected prediction algorithms.

**Figure 9:**
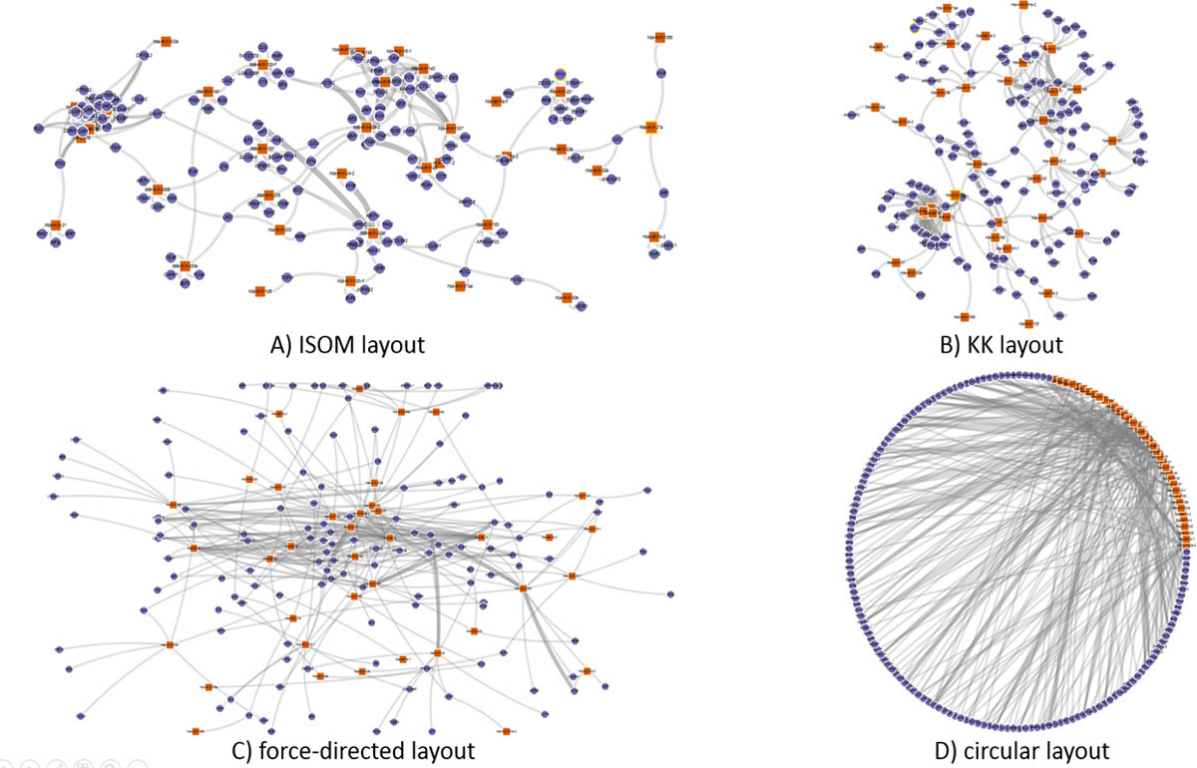
**Four node-link diagram layouts of *miRTarVis***.

**Figure 10:**
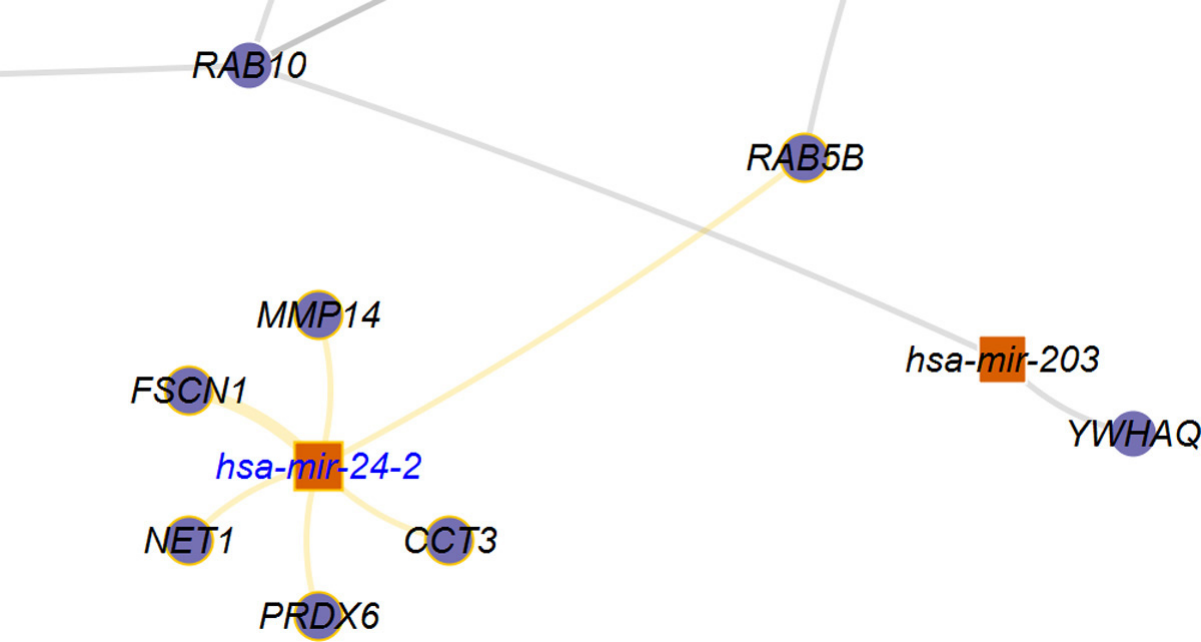
**Modified ISOM layout of *miRTarVis***. In this figure, among predicted targets of hsa-mir-24-2, mRNAs connected to hsa-mir-24-2 with a single link are placed around it. In the original ISOM layout, they were placed at the same position.

In the *visualize *menu, *miRTarVis *shows predicted miRNA-target interactions in node-link diagram or Treemaps (Figures 2 and 3). The node-link diagram in *miRTarVis *comes in one of four layouts: modified ISOM, KK, force-directed [18], and circular (Figure 9). miRNAs with common targets, which may indicate that their biological functions can be similar to one another, tend to be located closer in the modified ISOM and KK layouts. We modified the original ISOM layout such that the mRNAs connected to a given miRNA with a single link are placed around the miRNA (Figure 10). The width of a link in the node-link diagram represents the number of prediction algorithms that predict the corresponding miRNA-target pairs (Figures 2A and 3A).

**Figure 11:**
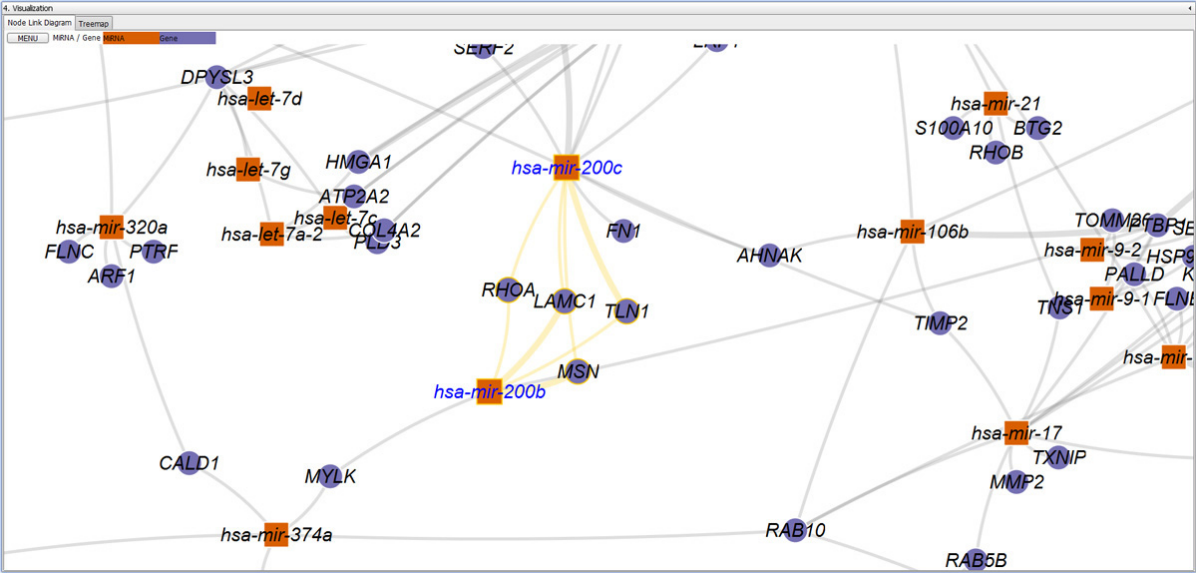
**Node-link diagram interaction of selection of multiple miRNAs**. hsa-mir-200c and hsa-mir-200b are selected, and *miRTarVis *highlights their common targets (PHOA, LAMC1, TLN1, and MSN).

The node-link diagram supports intuitive zooming in/out interaction by mouse wheel and panning interaction by dragging with the right mouse button. Double-clicking on a link shows a scatterplot between the corresponding miRNA and mRNA pair. When users click on a node in a node-link diagram, connected links and nodes are highlighted. If a user selects multiple miRNAs (clicking with the Shift key pressed), *miRTarVis *highlights their common targets (Figure 11). This feature is valuable because commonly targeted mRNA could play an important role in a miRNA-target network. Through a context menu, *miRTarVis *provides external links to miRBase [19] and miR2Disease [20] for miRNAs and to NCBI and GeneCards [21] for mRNAs. The ISOM and KK layouts of *miRTarVis *present an effective overview of miRNA regulatory network, but they have a common limitation of node occlusion with excessive nodes and links. Users can alleviate this problem by relocating a node manually (by dragging and moving nodes) to mend for a clean node-link diagram exported in their publication.

*miRTarVis *also provides gene enrichment analysis (provided by the Gene Ontology Consortium web service) of target genes by a miRNA. With accurate prediction, the biological functions of predicted targets of a miRNA tend to be similar. Through enrichment analysis, *miRTarVis *provides a convenient way for confirming the function of a miRNA and the validity of target prediction result.

### Implementation

*miRTarVis*, implemented using Java, runs on any systems with JRE version 1.7 or higher. In this study, *miRTarVis *used the first of the two methods presented by Kraskov et al. [22] for mutual information estimation. In contrast to conventional mutual information estimators using binning of variables, the method by Kraskov et al. used k-nearest neighbor distances to estimate mutual information, resulting in a better precision. We implemented the algorithm in Java and integrated it into *miRTarVis*.

The GenMiR++ algorithm, Bayesian inference analysis for miRNA target prediction, is originally implemented in Matlab. We converted it into Java and integrated it into *miRTarVis*. We validated our implementation by checking whether our result is the same as that of original Matlab implementation using the miRNA-mRNA expression profile data submitted by the authors of GenMiR++ (151 human miRNAs and 16,063 mRNAs across 88 tissues).

The MINE analysis finds highly associated variable pairs in multivariate dataset. It aims to improve generality (not limited to specific function types, i.e., linear or exponential) and equitability (similar score for equally noisy relationships of different types) of analysis. It calculates a maximal information coefficient score, which reflects the intensity of association between two variables. We implemented the MINE analysis algorithm [23] in Java and integrated it into *miRTarVis*.

*miRTarVis *embedded TargetScan databases. We downloaded a miRNA family table and a predicted conserved target information table and joined the two tables into one. The resulting table contains three attributes: miRBase ID, gene symbol, and species. *miRTarVis *has the resulting table that contains a set of conserved targets of all miRNAs for nine species (human, mouse, rat, rhesus, frog, dog, cow, chimpanzee, and chicken). *miRTarVis *also embedded databases from microRNA.org (microRNA.org). We downloaded target predictions with good mirSVR scores and conserved miRNA from the August 2010 release from the website and embedded it into *miRTarVis*.

## Results and discussion

As the rate of comorbid asthma and obesity increases, identifying mechanisms by which obesity affects asthma is critical. Our group reported that obese visceral adipocytes shed exosomes containing miRNAs that can up-regulate the expression of profibrotic signaling genes in the lung [24]. An important next step in our analyses was to define the set of lung mRNA responses to these adipocyte-derived exosomes. Prior standard approaches would have included generating a list of potential target mRNAs and prioritizing them for validation. miRTarVis presented us with a new opportunity to objectively define our target validation set of mRNAs using multiple in silico analyses.

Using airway fibroblasts (i.e., cells important in the development of lung fibrosis), we demonstrated the use of *miRTarVis *to define potential target mRNAs through which obesity can induce lung fibrosis in asthma. We used obese visceral adipocyte-derived exosomes (n = 4) that were previously tested for miRNA expression (Affymetrix microRNA 3.0 array). We coincubated these exosomes with human airway fibroblasts (from endobronchial biopsy tissue) from nonasthmatic and asthmatic donors (n = 1 each) for 24 hours. Fibroblasts were profiled for global mRNA expression.

One of the major advantages of *miRTarVis *is that it enables instantaneous application of multiple analytical algorithms to the data. Normalized, background-subtracted miRNA-mRNA expression profile data were imported into *miRTarVis *and filtered for a paired two-tailed t-test with p ≤ 0.05. Multiple prediction algorithms (i.e., Pearson correlation, MINE, GenMiR++, and TargetScan) were applied, and the top 1,000 negative correlations and top 100 opposite change direction were selected in each algorithm. The intersection among them could be efficiently identified through the filter step of *miRTarVis*.

**Figure 12:**
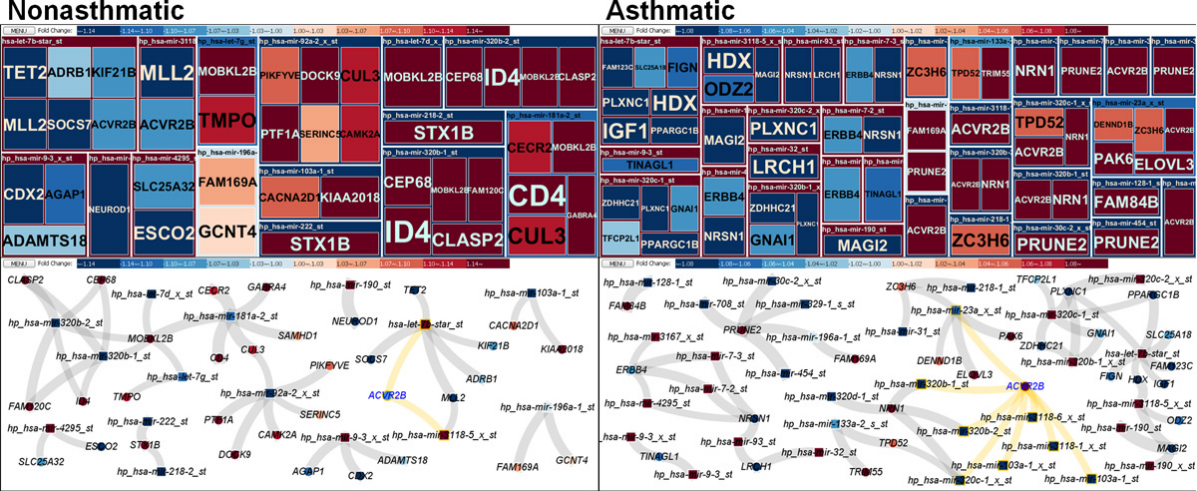
**Interrelations among miRNAs and mRNAs for nonasthmatic or asthmatic fibroblasts exposed to obese visceral exosomes**.

As shown in Table 1 we identified 45 miRNA-mRNA pairs (15 miRNAs / 33 mRNAs) for obese visceral exosomes and nonasthmatic fibroblasts and 61 miRNA-mRNA pairs (33 miRNAs / 27 mRNAs) for asthmatic fibroblasts. Our focus turned to ACVR2B (activin receptor, type IIB; myostatin and TGFβ receptor) as the only gene present in both datasets, that is, down-regulated in nonasthmatic fibroblasts (fold change [FC] = -1.18, p < 0.01) and up-regulated in asthmatic fibroblasts (FC = 1.31, p = 0.02). Figure 12 shows that obese visceral exosomal miRNAs targeting ACVR2B were up-regulated in nonasthmatic fibroblasts (i.e., hsa-let-7b-star_st [FC = 2.31, p = 0.027] and hp_hsa-mir-3118-5_x_st [FC = 2.16, p = 0.025]) and down-regulated in asthmatic fibroblasts (hp_hsa-mir-103a-1_st [FC = -2.47, p < 0.001], hp_hsa-mir-103a-1_x_st [FC = -2.18, p = 0.003], hp_hsa-mir-23a_x_st [FC = -1.08, p = 0.035], hp_hsa-mir-3118-1_x_st [FC = -1.53, p = 0.029], hp_hsa-mir-3118-6_x_st [FC = -1.56, p = 0.008], hp_hsa-mir-320b-1_st [FC = -2.41, p = 0.002], hp_hsa-mir-320b-2_st [FC = -1.51, p = 0.019], and hp_hsa-mir-320c-1_x_st [FC = -2.37, p = 0.004]). qRT-PCR confirmed ACVR2B down-regulation in nonasthmatic fibroblasts (FC = 0.26, 95% confidence interval = [0.26, 0.78]) and up-regulation in asthmatic fibroblasts (FC = 3.21, [3.21, 6.72]).

**Table 1:** miRNA-mRNA pairs for obese visceral exosomes and nonasthmatic or asthmatic fibroblast.

Non-asthmatic							Asthmatic						
**microRNA**	**mRNA**	**Correlation**	**GenMiR++**	**MINE**	**TargetScan**	microRNA.org	**microRNA**	**mRNA**	**Correlation**	**GenMiR++**	**MINE**	**TargetScan**	microRNA.org

hp_hsa-mir-3118-5_x_st	ACVR2B	TRUE	TRUE	FALSE	TRUE	FALSE	hp_hsa-mir-3118-1_x_st	ACVR2B	TRUE	TRUE	FALSE	TRUE	FALSE

hsa-let-7b-star_st	ACVR2B	TRUE	TRUE	FALSE	TRUE	FALSE	hp_hsa-mir-320b-1_st	ACVR2B	TRUE	TRUE	FALSE	TRUE	FALSE

hp_hsa-mir-9-3_x_st	ADAMTS18	TRUE	TRUE	FALSE	TRUE	TRUE	hp_hsa-mir-103a-1_st	ACVR2B	TRUE	TRUE	FALSE	TRUE	FALSE

hsa-let-7b-star_st	ADRB1	TRUE	TRUE	FALSE	TRUE	TRUE	hp_hsa-mir-3118-6_x_st	ACVR2B	TRUE	TRUE	FALSE	TRUE	FALSE

hp_hsa-mir-9-3_x_st	AGAP1	TRUE	TRUE	FALSE	TRUE	TRUE	hp_hsa-mir-320b-2_st	ACVR2B	TRUE	TRUE	FALSE	TRUE	FALSE

hp_hsa-mir-103a-1_st	CACNA2D1	TRUE	TRUE	FALSE	TRUE	FALSE	hp_hsa-mir-320c-1_x_st	ACVR2B	TRUE	TRUE	FALSE	TRUE	FALSE

hp_hsa-mir-92a-2_x_st	CAMK2A	TRUE	TRUE	FALSE	TRUE	TRUE	hp_hsa-mir-23a_x_st	ACVR2B	TRUE	TRUE	FALSE	TRUE	FALSE

hp_hsa-mir-181a-2_st	CD4	TRUE	TRUE	TRUE	TRUE	TRUE	hp_hsa-mir-103a-1_x_st	ACVR2B	TRUE	TRUE	FALSE	TRUE	FALSE

hp_hsa-mir-9-3_x_st	CDX2	TRUE	TRUE	FALSE	TRUE	TRUE	hp_hsa-mir-23a_x_st	DENND1B	TRUE	TRUE	FALSE	TRUE	FALSE

hp_hsa-mir-181a-2_st	CECR2	TRUE	TRUE	TRUE	TRUE	TRUE	hp_hsa-mir-23a_x_st	ELOVL3	TRUE	TRUE	FALSE	TRUE	TRUE

hp_hsa-mir-320b-2_st	CEP68	TRUE	TRUE	FALSE	TRUE	FALSE	hp_hsa-mir-4295_st	ERBB4	TRUE	TRUE	FALSE	TRUE	FALSE

hp_hsa-mir-320b-1_st	CEP68	TRUE	TRUE	FALSE	TRUE	FALSE	hp_hsa-mir-3167_x_st	ERBB4	TRUE	TRUE	FALSE	TRUE	FALSE

hp_hsa-mir-320b-2_st	CLASP2	TRUE	TRUE	FALSE	TRUE	TRUE	hp_hsa-mir-7-2_st	ERBB4	TRUE	TRUE	FALSE	TRUE	TRUE

hp_hsa-mir-320b-1_st	CLASP2	TRUE	TRUE	FALSE	TRUE	TRUE	hp_hsa-mir-7-3_st	ERBB4	TRUE	TRUE	FALSE	TRUE	TRUE

hp_hsa-mir-92a-2_x_st	CUL3	TRUE	TRUE	FALSE	TRUE	FALSE	hsa-let-7b-star_st	FAM123C	TRUE	TRUE	FALSE	TRUE	TRUE

hp_hsa-mir-181a-2_st	CUL3	TRUE	TRUE	TRUE	TRUE	TRUE	hp_hsa-mir-196a-1_st	FAM169A	TRUE	TRUE	FALSE	TRUE	TRUE

hp_hsa-mir-92a-2_x_st	DOCK9	TRUE	TRUE	FALSE	TRUE	TRUE	hp_hsa-mir-128-1_st	FAM84B	TRUE	TRUE	FALSE	TRUE	TRUE

hp_hsa-mir-4295_st	ESCO2	TRUE	TRUE	FALSE	TRUE	FALSE	hsa-let-7b-star_st	FIGN	TRUE	TRUE	FALSE	TRUE	TRUE

hp_hsa-mir-320b-2_st	FAM120C	TRUE	TRUE	FALSE	TRUE	TRUE	hp_hsa-mir-320c-1_st	GNAI1	TRUE	TRUE	FALSE	TRUE	TRUE

hp_hsa-mir-320b-1_st	FAM120C	TRUE	TRUE	FALSE	TRUE	TRUE	hp_hsa-mir-320b-1_x_st	GNAI1	TRUE	TRUE	FALSE	TRUE	TRUE

hp_hsa-mir-196a-1_st	FAM169A	TRUE	TRUE	FALSE	TRUE	TRUE	hp_hsa-mir-3118-5_x_st	HDX	TRUE	TRUE	FALSE	TRUE	FALSE

hp_hsa-mir-181a-2_st	GABRA4	TRUE	TRUE	TRUE	TRUE	FALSE	hsa-let-7b-star_st	HDX	TRUE	TRUE	FALSE	TRUE	FALSE

hp_hsa-mir-196a-1_st	GCNT4	TRUE	TRUE	FALSE	TRUE	TRUE	hsa-let-7b-star_st	IGF1	TRUE	TRUE	FALSE	TRUE	FALSE

hp_hsa-mir-320b-2_st	ID4	TRUE	TRUE	FALSE	TRUE	FALSE	hp_hsa-mir-32_st	LRCH1	TRUE	TRUE	FALSE	TRUE	TRUE

hp_hsa-mir-320b-1_st	ID4	TRUE	TRUE	FALSE	TRUE	FALSE	hp_hsa-mir-93_st	LRCH1	TRUE	TRUE	FALSE	TRUE	TRUE

hp_hsa-mir-103a-1_st	KIAA2018	TRUE	TRUE	FALSE	TRUE	FALSE	hp_hsa-mir-3118-5_x_st	MAGI2	TRUE	TRUE	FALSE	TRUE	FALSE

hsa-let-7b-star_st	KIF21B	TRUE	TRUE	FALSE	TRUE	FALSE	hp_hsa-mir-190_st	MAGI2	TRUE	TRUE	FALSE	TRUE	TRUE

hp_hsa-mir-3118-5_x_st	MLL2	TRUE	TRUE	FALSE	TRUE	FALSE	hp_hsa-mir-190_x_st	MAGI2	TRUE	TRUE	FALSE	TRUE	TRUE

hsa-let-7b-star_st	MLL2	TRUE	TRUE	FALSE	TRUE	TRUE	hp_hsa-mir-320d-1_st	NRN1	TRUE	TRUE	FALSE	TRUE	TRUE

hp_hsa-let-7d_x_st	MOBKL2B	TRUE	TRUE	FALSE	TRUE	FALSE	hp_hsa-mir-320b-1_st	NRN1	TRUE	TRUE	FALSE	TRUE	TRUE

hp_hsa-mir-320b-2_st	MOBKL2B	TRUE	TRUE	FALSE	TRUE	FALSE	hp_hsa-mir-320b-2_st	NRN1	TRUE	TRUE	FALSE	TRUE	TRUE

hp_hsa-mir-320b-1_st	MOBKL2B	TRUE	TRUE	FALSE	TRUE	FALSE	hp_hsa-mir-320c-1_x_st	NRN1	TRUE	TRUE	FALSE	TRUE	TRUE

hp_hsa-let-7g_st	MOBKL2B	TRUE	TRUE	FALSE	TRUE	FALSE	hp_hsa-mir-4295_st	NRSN1	TRUE	TRUE	FALSE	TRUE	FALSE

hp_hsa-mir-181a-2_st	MOBKL2B	TRUE	TRUE	TRUE	TRUE	FALSE	hp_hsa-mir-93_st	NRSN1	TRUE	TRUE	FALSE	TRUE	TRUE

hp_hsa-mir-190_st	NEUROD1	TRUE	TRUE	TRUE	TRUE	TRUE	hp_hsa-mir-7-2_st	NRSN1	TRUE	TRUE	FALSE	TRUE	TRUE

hp_hsa-mir-92a-2_x_st	PIKFYVE	TRUE	TRUE	FALSE	TRUE	TRUE	hp_hsa-mir-7-3_st	NRSN1	TRUE	TRUE	FALSE	TRUE	TRUE

hp_hsa-mir-92a-2_x_st	PTF1A	TRUE	TRUE	FALSE	TRUE	TRUE	hp_hsa-mir-3118-5_x_st	ODZ2	TRUE	TRUE	FALSE	TRUE	FALSE

hp_hsa-mir-181a-2_st	SAMHD1	TRUE	TRUE	FALSE	TRUE	TRUE	hp_hsa-mir-23a_x_st	PAK6	TRUE	TRUE	FALSE	TRUE	TRUE

hp_hsa-mir-92a-2_x_st	SERINC5	TRUE	TRUE	FALSE	TRUE	FALSE	hp_hsa-mir-320c-2_x_st	PLXNC1	TRUE	TRUE	TRUE	TRUE	TRUE

hp_hsa-mir-4295_st	SLC25A32	TRUE	TRUE	FALSE	TRUE	FALSE	hp_hsa-mir-320c-1_st	PLXNC1	TRUE	TRUE	FALSE	TRUE	TRUE

hsa-let-7b-star_st	SOCS7	TRUE	TRUE	FALSE	TRUE	TRUE	hp_hsa-mir-320b-1_x_st	PLXNC1	TRUE	TRUE	FALSE	TRUE	TRUE

hp_hsa-mir-222_st	STX1B	TRUE	TRUE	TRUE	TRUE	FALSE	hsa-let-7b-star_st	PLXNC1	TRUE	TRUE	FALSE	TRUE	FALSE

hp_hsa-mir-218-2_st	STX1B	TRUE	TRUE	FALSE	TRUE	FALSE	hp_hsa-mir-320c-1_st	PPARGC1B	TRUE	TRUE	FALSE	TRUE	FALSE

hsa-let-7b-star_st	TET2	TRUE	TRUE	FALSE	TRUE	FALSE	hsa-let-7b-star_st	PPARGC1B	TRUE	TRUE	FALSE	TRUE	TRUE

hp_hsa-let-7g_st	TMPO	TRUE	TRUE	FALSE	TRUE	FALSE	hp_hsa-mir-30c-2_x_st	PRUNE2	TRUE	TRUE	FALSE	TRUE	TRUE

							hp_hsa-mir-708_st	PRUNE2	TRUE	TRUE	FALSE	TRUE	TRUE

							hp_hsa-mir-329-1_s_st	PRUNE2	TRUE	TRUE	FALSE	TRUE	TRUE

							hp_hsa-mir-454_st	PRUNE2	TRUE	TRUE	FALSE	TRUE	TRUE

							hp_hsa-mir-196a-1_st	PRUNE2	TRUE	TRUE	FALSE	TRUE	TRUE

							hsa-let-7b-star_st	SLC25A18	TRUE	TRUE	FALSE	TRUE	TRUE

							hp_hsa-mir-320c-1_st	TFCP2L1	TRUE	TRUE	FALSE	TRUE	FALSE

							hp_hsa-mir-9-3_st	TINAGL1	TRUE	TRUE	FALSE	TRUE	TRUE

							hp_hsa-mir-9-3_x_st	TINAGL1	TRUE	TRUE	FALSE	TRUE	TRUE

							hp_hsa-mir-133a-2_s_st	TPD52	TRUE	TRUE	FALSE	TRUE	FALSE

							hp_hsa-mir-320c-1_x_st	TPD52	TRUE	TRUE	FALSE	TRUE	FALSE

							hp_hsa-mir-133a-2_s_st	TRIM55	TRUE	TRUE	FALSE	TRUE	TRUE

							hp_hsa-mir-218-1_st	ZC3H6	TRUE	TRUE	FALSE	TRUE	TRUE

							hp_hsa-mir-31_st	ZC3H6	TRUE	TRUE	FALSE	TRUE	FALSE

							hp_hsa-mir-23a_x_st	ZC3H6	TRUE	TRUE	FALSE	TRUE	FALSE

							hp_hsa-mir-320c-1_st	ZDHHC21	TRUE	TRUE	FALSE	TRUE	TRUE

							hp_hsa-mir-320b-1_x_st	ZDHHC21	TRUE	TRUE	FALSE	TRUE	TRUE

In summary, *miRTarVis *analyses quickly and inexpensively identified a biologically relevant mRNA target for adipocyte-derived exosomal miRNAs. This target, ACVR2B, is down-regulated in nonasthmatic fibroblasts and up-regulated in asthmatic fibroblasts, suggesting that obese visceral adipocyte-derived exosomes regulate airway fibroblast gene expression and that these cells respond differently to the exosomes depending on disease state. *miRTarVis *enabled this novel mechanistic discovery by which adiposity may increase lung fibrosis in asthma.

This case study could reasonably serve as a proof of concept showing the efficacy of miRTarVis, but more rigorous case studies with more participants are necessary to generalize the findings of this case study.

We also found a limitation in our node-link diagram visualization in this case study. The layout of the node-link diagram in *miRTarVis *(Figure 12) changes (often significantly) whenever the user changes the filtering or prediction parameters. Following the change and comparing the two different networks were difficult for the user because *miRTarVis *does not place identical miRNA or mRNA nodes in the same position. This situation was caused by the random and iterative nature of the layout algorithms. In future work, we will modify the layout algorithm so that nodes maintain their position as much as possible when users change the parameters.

## Conclusions

We introduced *miRTarVis*, an interactive visual analysis tool for miRNA-mRNA expression profile data. We defined a representative analysis pipeline for the data and designed mirTarVis to support the analysis pipeline based on a foldable accordion metaphor. miRTarVis integrates various miRNA target prediction algorithms, including a novel one using the MINE analysis, into the analysis pipeline. miRTarVis shows the resulting miRNA target network using an interactive Treemap and node-link diagram with improved layout algorithms.

We conducted a case study to prove the efficacy of *miRTarVis*. We analyzed a miRNA-mRNA expression profile data of asthma patients and found a potentially novel mechanism by which adiposity increases fibrosis in asthma.

As a future work, we will improve *miRTarVis *by integrating TFs, which regulate gene expression with miRNAs, into the analysis procedure of *miRTarVis*. We will conduct further study on visualization techniques for the gene expression regulatory network and integrate new visualization techniques into *miRTarVis*. We will also research on a new interaction technique to solve a problem of the Treemap, namely, the lack of the visual cue to show common targets of a miRNA. We will also conduct a controlled user study to a miRNA researcher to verify the usefulness of our tool more objectively.

## List of abbreviations

miRNA (microRNA), TCGA (The Cancer Genome Atlas)

## Competing interests

The authors declare that they have no competing interests.

## Authors' contributions

DJ, BK, MG, EH, and JS designed this study and participated in ideation and design of visualizations and interactions for the tool. DJ implemented the tool. MG, RF, and EH tested the tool and suggested improvements. RF participated in the case study.

## Declarations

The publication fees and Open Access expenses for this article were funded by an academic activity support program of Seoul National University.

This article has been published as part of *BMC Proceedings *Volume 9 Supplement 6, 2015: Proceedings of the 5th Symposium on Biological Data Visualization: Part 2. The full contents of the supplement are available online at http://www.biomedcentral.com/bmcproc/supplements/9/S6.
